# Molecular Diagnostics of Banana Fusarium Wilt Targeting *Secreted-in-Xylem* Genes

**DOI:** 10.3389/fpls.2019.00547

**Published:** 2019-05-31

**Authors:** Lilia C. Carvalhais, Juliane Henderson, Vivian A. Rincon-Florez, Cecilia O’Dwyer, Elizabeth Czislowski, Elizabeth A. B. Aitken, André Drenth

**Affiliations:** ^1^Queensland Alliance for Agriculture and Food Innovation, Centre for Horticultural Science, Ecosciences Precinct, The University of Queensland, Brisbane, QLD, Australia; ^2^School of Agriculture and Food Sciences, The University of Queenslandxy3Saint Lucia, QLD, Australia

**Keywords:** tropical race 4, molecular diagnostics, Panama disease, *SIX* genes, plant pathogens, *Fusarium oxysporum*

## Abstract

Fusarium wilt is currently spreading in banana growing regions around the world leading to substantial losses. The disease is caused by the fungus *Fusarium oxysporum* f. sp. *cubense* (*Foc*), which is further classified into distinct races according to the banana varieties that they infect. Cavendish banana is resistant to *Foc* race 1, to which the popular Gros Michel subgroup succumbed last century. Cavendish effectively saved the banana industry, and became the most cultivated commercial subgroup worldwide. However, *Foc* tropical race 4 (TR4) subsequently emerged in Southeast Asia, causing significant yield losses due to its high level of aggressiveness to cultivars of Cavendish, and other commonly grown cultivars. Preventing further spread is crucially important in the absence of effective control methods or resistant market-acceptable banana cultivars. Implementation of quarantine and containment measures depends on early detection of the pathogen through reliable diagnostics. In this study, we tested the hypothesis that *secreted in xylem* (*SIX*) genes, which currently comprise the only known family of effectors in *F. oxysporum*, contain polymorphisms to allow the design of molecular diagnostic assays that distinguish races and relevant VCGs of *Foc*. We present specific and reproducible diagnostic assays based on conventional PCR targeting *SIX* genes, using as templates DNA extracted from pure *Foc* cultures. Sets of primers specifically amplify regions of: *SIX6* in *Foc* race 1, *SIX1* gene in TR4, *SIX8* in subtropical race 4, *SIX9*/*SIX10* in *Foc* VCG 0121, and *SIX13* in *Foc* VCG 0122. These assays include simplex and duplex PCRs, with additional restriction digestion steps applied to amplification products of genes *SIX1* and *SIX13*. Assay validations were conducted to a high international standard including the use of 250 *Fusarium* spp. isolates representing 16 distinct *Fusarium* species, 59 isolates of *F. oxysporum*, and 21 different vegetative compatibility groups (VCGs). Tested parameters included inter and intraspecific analytical specificity, sensitivity, robustness, repeatability, and reproducibility. The resulting suite of assays is able to reliably and accurately detect R1, STR4, and TR4 as well as two VCGs (0121 and 0122) causing Fusarium wilt in bananas.

## Introduction

Commercial banana production is under serious threat worldwide. A destructive disease caused by the fungus *Fusarium oxysporum* f. sp. *cubense* tropical race 4 (TR4) is spreading rapidly throughout the banana growing regions around the globe (Ordonez et al., 2015; Zheng et al., 2018). The soilborne nature of this pathogen is one of the main reasons why eradication and containment of TR4 are very challenging. In the absence of effective disease resistance in market-accepted banana varieties, one of the remaining measures to avoid further losses is to prevent spread through early detection with reliable diagnostics, and subsequent containment of new incursions.

Tropical race 4 was first identified in samples obtained from Sumatra, Indonesia, in 1992 (Ploetz, 1994; Ploetz, 2004). However, reports of infected Cavendish banana (*Musa* spp. AAA genome group) date from nearly 30 years before in Taiwan (Su et al., 1977; Ploetz, 1994). Since its emergence, TR4 has not only decimated the Cavendish banana industry in Taiwan, but has been making its way through Southeast Asia in China, Indonesia, Malaysia, Philippines, Laos, Myanmar, Vietnam, and Pakistan (Ordonez et al., 2015, 2016; Chittarath et al., 2018; Hung et al., 2018; Zheng et al., 2018). Intercontinental spread has also occurred with reports of the pathogen in Australia, Jordan, Lebanon, Oman, and Mozambique (Butler, 2013; García-Bastidas et al., 2014; Ploetz, 2015b; Dita et al., 2018).

The present situation of Fusarium wilt is familiar: another race of the causal agent (race 1 or R1) triggered a devastating epidemic in the banana growing regions of Central America in the first half of last century (Stover, 1990; Ploetz, 2005). It was “widely regarded as one of the most destructive plant diseases in recorded history” (Moore et al., 1995). A major commercial subgroup at the time, “Gros Michel” (*Musa acuminata* AAA genome group), was highly susceptible to R1 (Stover, 1962). The banana industry could only recover because cultivars belonging to the Cavendish subgroup were found to be resistant to R1 (Ploetz, 1994).

Resistance to Fusarium wilt R1, high productivity, the use of temperature-controlled transportation in boxes, and consumer acceptance turned Cavendish into the main subgroup used worldwide for export (Ploetz, 2005). Other varieties of banana are fundamental to the subsistence of households of many developing countries with low incomes, either as a staple or as a cash crop (FAOSTAT, 2018). With predictions of many of these countries being the main contributors to population growth (Gerland et al., 2014), the protection of banana production is essential to safeguard not only one of the most popularly consumed and exported fruits globally, but also livelihoods of millions of people (FAOSTAT, 2018).

To facilitate reference to groups that infect specific plant species, *F. oxysporum* has been sub-divided into *formae speciales* (Armstrong, 1981). However, given that not all varieties of the same plant species are necessarily susceptible to a particular *forma specialis*, these are further classified into races, e.g., *F. oxysporum* f. sp. *lycopersici* race 1 infects a specific group of tomato varieties (Alexander, 1945). The term *forma specialis cubense* is utilized to delineate those populations that can cause disease on banana. Nevertheless, the race classification can be inconsistent as surprisingly little work focused on identifying the host specificity of this pathogen (Ploetz, 2015a). In general, the cultivars within the subgroup Gros Michel (AAA), Pisang Awak (ABB) and others that belong to the AAB genomic group (e.g., Maqueno, Silk, and Pome) are susceptible to R1 (Stover and Buddenhagen, 1986; Stover and Simmonds, 1987; Stover, 1990). The susceptibility of banana varieties to races 2 and 4 is still unclear as insufficient variety trials have been systematically conducted for these two races. Race 4 was further divided into two groups, TR4 and subtropical race 4 (STR4). Lower temperatures are typically associated with STR4 infection and disease progression in cultivars of the Cavendish subgroup in subtropical regions, while TR4 is virulent to these varieties in all environments (Ploetz, 2005). Reclassification of TR4 into the new species *Fusarium odoratissimum* was proposed based on the genetic diversity of *Foc* isolates in the Indonesian centre of origin (Maryani et al., 2019).

An additional classification commonly used for the fungi causing Fusarium wilt is based on vegetative compatibility. Strains are classified into the same vegetative compatibility group (VCG) when they are able to anastomose and form a stable heterokaryon with each other (Leslie, 1993). This system reflects well the similarity of strains based on phenotypical traits (Caten and Jinks, 1966); however, genetic relatedness between different groupings cannot be inferred as mutations in the *vic* loci can lead to vegetative incompatibility even in closely related isolates (Bentley et al., 1998). Although different races can be associated with most VCGs, there are several limitations in the use of VCGs to support race classifications. Various VCGs can be associated with the same race, and evolutionary relationships cannot be taken into consideration (Ordonez et al., 2015). For example, VCG 0126 exhibits a closer phylogenetic affiliation to race 4 and, similarly to isolates classified into this race, have the ability to produce odorous aldehydes on media (Moore et al., 1991; Czislowski et al., 2017). Nonetheless, they are considered race 1 due to the host range on which it is able to cause disease (Pegg et al., 1994).

Breeding of new banana cultivars has been attempted, but until now has only had very limited success. Progress in banana breeding has been hampered by many factors, which include lack of germplasm evaluation, lack of fundamental genetic studies of diploid plants to identify characteristics, as well as lack of a long-term commitment to fund breeding. Besides, the occurrence of varying ploidy levels in cultivated varieties (which are polyploid) and the need for diploids for crosses, parthenocarpy, sterility, poor seed set, germination, and survival have hampered progress in banana breeding programs (Pillay and Tenkouano, 2011). Nonetheless, the growing impact of TR4 and other diseases such as black leaf streak (black Sigatoka, caused by the fungus *Pseudocercospora fijiensis*) and several improvements in breeding processes have yielded a renewed interest in this area (Pillay and Tenkouano, 2011; Li et al., 2015), while transgenic approaches to obtain TR4-resistant Cavendish are undergoing field-testing (Dale et al., 2017).

Once banana plants are infected, there is no effective treatment for Fusarium wilt. Fungicides or soil fumigation are ineffective to control or eradicate this disease (Ploetz, 2015b). Thus, in the absence of effective resistance or control methods, one of the remaining ways to manage this disease is preventing further spread and ensuring rapid containment of new infected plants. For this, early identification of infected plants combined with sensitive, accurate and robust diagnostic methods to detect incursions at an early stage are paramount. Diagnosing the specific VCGs and races is required as similar early symptoms caused by distinct strains are observed in various locally grown non-Cavendish and commercialized banana varieties (Karangwa et al., 2016). Diagnostics based solely on morphology is not reliable to differentiate distinct *Foc* and non-pathogenic strains of *F. oxysporum* (Pérez Vicente et al., 2014). Several molecular diagnostics methods have been developed, which mostly rely on core genomic regions and hence are unlikely to be closely linked or associated with pathogenic characteristics of different races. A molecular diagnostic method based on the intergenic spacer region (IGS) of the nuclear ribosomal gene cluster of *Foc* is widely used and is efficient for detecting TR4 (Dita et al., 2010). However, this assay was not designed to detect other strains that are closely related to TR4 and are also able to infect Cavendish, such as those affiliated to VCG 0121 and VCG 0122 (Ordonez et al., 2015; Czislowski et al., 2017; Mostert et al., 2017). Another study conducted by Lin et al. (2009) reported primers that are able to detect all race 4 strains, and hence can detect strains assigned to VCG 0121 and VCG 0122. However, these primers are unable to distinguish TR4 and STR4. Despite being closely related to TR4 VCGs 01213/16 and being able to attack Cavendish varieties in the tropics, the classification of R4 VCGs 0121 and 0122 as TR4 or STR4 is still a matter of debate (Moore et al., 1993; Bentley et al., 1998; Buddenhagen, 2009). Some of the isolates belonging to VCGs 0121 and 0122 have been reported to infect Cavendish causing symptoms that were less severe than those caused by TR4 isolates (Buddenhagen, 2009), which suggests that predisposing conditions may need to be in place for symptoms to be expressed. Therefore, we opted for classifying here VCGs 0121 and 0122 as R4, without further sub-classifications as tropical or subtropical. *Foc* strains affiliated to these VCGs can cause disease in Cavendish (Mostert et al., 2017), thus having molecular tools available to detect VCG 0121 and VCG 0122 is especially beneficial for countries reliant on banana cultivation under subtropical conditions.

A common target for diagnostics is genes that encode proteins that are strongly correlated with virulence (de Sain and Rep, 2015). In *F. oxysporum*-infected tomato, some of these proteins have been detected in the xylem sap and named Secreted-in-Xylem (SIX) (Rep et al., 2004; Houterman et al., 2007; Gawehns et al., 2014). When these proteins or other molecules (e.g., secondary metabolites and small RNAs) are associated with processes in the host in favor of colonization by the pathogen and disease progression, they are known as effectors (Weiberg et al., 2013; de Sain and Rep, 2015). Homologs of the *SIX* genes are found across different *formae speciales* and their profile can be used to distinguish distinct *formae speciales*, races and isolates (Lievens et al., 2009; Chakrabarti et al., 2011; van Dam et al., 2016; Czislowski et al., 2017). *SIX* gene profiling has been successfully used to distinguish the three races in *F. oxysporum* f. sp. *lycopersici* (Lievens et al., 2009). The mode of action of these proteins in the pathogenicity process remains to be elucidated, but some studies have suggested an effect on plant defense signaling (Thatcher et al., 2012; Kazan and Lyons, 2014).

Diagnostic tools to identify races associated with banana Fusarium wilt are needed to allow early detection of new incursions and distinguish isolates infecting varieties that are generally more susceptible to a wider range of *Fusarium* (e.g., Lady Finger). However, poor assay validation often leads to a huge gap between development and implementation of diagnostic methods (Chilvers, 2012). Evidence for the specificity and reliability of diagnostic assays is required and therefore validation needs to be conducted according to rigorous standards.

The gene *SIX8* has been previously shown to contain enough variation to differentiate *Foc* race 4 from the races 1 and 2 as well as STR4 from TR4 through a conventional PCR (Fraser-Smith et al., 2014). However, only three non-pathogenic isolates of *F. oxysporum* were included in the screening, and the detection of TR4 was based on the absence of an amplification product. Diagnosing a disease based on the absence of an amplification product is unreliable because the absence of a pathogen would provide the same outcome. In addition, basic validation parameters such as sensitivity and robustness cannot be assessed if there is no amplicon for the assessment of positive diagnostics. Therefore, the overall objective of our study was to assess whether sequence variation in *SIX* genes of *Fusarium* races could be used to develop a series of assays that can detect relevant VCGs and races of Fusarium wilt of banana. We specifically sought to address whether: (1) *SIX* genes exhibit specific sequences that are conserved within each race; (2) there are sufficient single nucleotide polymorphisms (SNPs) across *SIX* gene homologs to enable the design of primers which target unique sequences within the above races; (3) the primers targeting specific *SIX* genes homologs can be used in PCR assays to reach a level of specificity, sensitivity, robustness and repeatability that meets international standards for diagnostic assays; and (4) our proposed assays can detect all races and VCGs mentioned above and outperform popularly used molecular diagnostic tests previously designed to detect R4 (STR4 and TR4).

Reliable detection of different races and VCGs of *Fusarium* will enable decision-making by the banana industry stakeholders.

## Materials and Methods

Regions in the *SIX* genes conserved within races of *Foc* were identified by analyzing sequences generated by Czislowski et al. (2017), which are available in the National Center for Biotechnology Information database^1^ (NCBI Resource Coordinators, 2017), under accessions KX434886-KX435052. These sequences had been generated by whole-genome sequencing (WGS) analysis followed by *Foc-SIX* specific PCR to characterize the diversity and evolution of the *SIX* genes in a collection of 89 isolates representing 23 genetic lineages of *Foc* (Czislowski et al., 2017). Based on the data of Czislowski et al. (2017), it was possible to identify in this current study an exclusive *SIX* gene homolog for each race of *Foc*. Each of these exclusive homologs was present in a different *SIX* gene. Consensus sequences of these *SIX* gene homologs were aligned for the identification of conserved regions within races of *Foc*. Alignments were performed either in Geneious (v10.0.9) (Kearse et al., 2012) or Clustal Omega^2^ (Sievers and Higgins, 2014). SNP-rich regions within the distinct *SIX* genes were then targeted for race-specific primer design, as described in the following sections.

### Primer Design

Sets of primers were designed either to anneal to SNP-rich regions within the targeted *SIX* gene or to anneal to regions flanking a specific SNP that contained a sequence which included a restriction enzyme site. Details of races, VCGs, target *SIX* gene homologs, primer sequences, and annealing temperatures are listed in Table 1. The strategy for identifying TR4 isolates was based on the exclusive presence of the homolog “a” of *SIX1* in TR4 (Czislowski et al., 2017). For the design of specific primers to detect TR4 isolates, full-length sequences from all *SIX1* gene homologs (“a” to “i”) were aligned using the software Clustal Omega^2^. The primer set SIX1_266 (Table 1) was designed to anneal to regions which were rich in SNPs that were exclusive to the *SIX1* gene homologs “a,” “b,” or “c.” These gene homologs are unique to TR4, R4 VCG 0121 and R4 VCG 0122, respectively (Czislowski et al., 2017). The 266 bp-product amplified by these primers contains a unique recognition site for the restriction enzyme HpyAV (New England BioLabs), which is present in *SIX1* homolog “a” and absent in homologs “b” and “c.” After restriction digestion, two DNA fragments are predicted to be generated, one with 124 bp and the other with 142 bp.

**Table 1:** Primer sequences designed in this study based on *SIX* gene sequences generated in Czislowski et al. (2017).

Race	VCG	Targeted	Primer	Sequence	Primer	Product	Anneal.	Restriction	Restriction
		*SIX* gene	name	(5′ -> 3′)	annealing	length	temperature	digestion	enzyme
					position	(bp)	(°C)		recognition
					within gene				site
All *Foc*	All	*SIX9*a	SIX9_Foc_F	ATCGCTGAAGCCCAGAACAA	46–65291–305	260	58	No	N/A
			SIX9_Foc_R	TTCTGTCCGTCGATCGTTCC					
R1	0123, 01210, 01217, 01218, 0124, 0124/5, 0124/22, 0125, 0128, 01220	*SIX6*b	SIX6b_210_F	ACGCTTCCC**A**ATACCG**T**CTGT	181–201371–390	210	55	No	N/A
			SIX6b_210_R	AAG**T**T**G**G**T**G**AGT**A**TC**AA**T**GC					
TR4	01213/16	*SIX1*a	SIX1a_266_F	GTGACCAGAACTTGCCCACA	442–461689–707	266	55	HpyAV, 124 bp/142 bp	**CCTTC**(N)_6_
			SIX1a_266_2_R	CTTTGATAAGCACCATC**AA**					
STR4	0120, 0120/15, 0129, 01211, 01215 0126^∗^	*SIX8*b	SIX8b_206_F	GCCTGCATAACAGGTGC**C**GG**T**	267–287448–472	206	62	No	N/A
			SIX8b_206_R	**T**TC**C**TCACCTCACCCGGCA**G**GAT**TC**					
R4^#^	0121	*SIX10*a	SIX10a_309_F	**CCACTGGCACCAAAGACTTG**	62–81351–370	309	58	No	N/A
			SIX10a_309_R, to be used as duplex in combination with SIX9_Foc	**CGATGCGGAGTACTGGTTGA**					
R4^#^	0122	*SIX13*c	SIX13c_343_F	CAGCCTCCTAGCGTCGAAAA	91–110414–433	343	57	EagI102 bp/241 bp	**C**GGC**C**G
			SIX13c_343_R	CCGTGATGGGGT**A**CGTTGTA					


*Nucleobases in the primer sequences represented **in bold** correspond to polymorphic sites in the target gene homolog. ‘N/A’ stands for not applicable.*

*^∗^The race of VCG 0126 is arguable as isolates are phenotypically and genetically similar to race 4 (e.g., ability to synthesize odorous aldehydes on media) (Moore et al., 1991; Dale et al., 2017). However, there is limited evidence to suggest that VCG 0126 is capable of infecting Cavendish and is therefore often considered race 1 based on its host range (Pérez Vicente et al., 2014).*

*^#^The classification of VCGs 0121 and 0122 as tropical or subtropical race 4 is arguable. Isolates of these VCGs are not as aggressive as those of VCG 01213/16 on Cavendish banana, which suggests that predisposing conditions may need to be in place for symptoms to be expressed. We thus opted for classifying these VCGs as R4, abstain from giving a further sub-classification and point out here that this is a matter of debate which needs further clarification.*

A different approach was adopted to detect R4 VCG 0121 and R4 VCG 0122. To specifically detect R4 VCG 0121, primers were designed to target the *SIX10* gene which is present exclusively in this VCG (Czislowski et al., 2017) using Primer-BLAST^3^. A 309 bp-product is predicted to be amplified with this primer set. However, we obtained the same amplicon for several *F. oxysporum* strains isolated from crops other than banana (data not shown). We thus opted for the use of a duplex PCR combining the primer set that targets *SIX10* in combination with the *SIX9* gene that is conserved in all isolates of *Foc* (Czislowski et al., 2017). The primer set SIX9_Foc was designed to amplify a 260-bp product targeting the homolog “a” of *SIX9* using the software Primer3 (Untergasser et al., 2012).

To detect VCG 0122, primers were designed to amplify a 343-bp region of the gene *SIX13* that contains a restriction site exclusive to this VCG recognized by the restriction enzyme EagI (New England Biolabs). A restriction digestion step after amplification was necessary as there was insufficient sequence variation to design primers that would amplify only the homolog “c” of *SIX13* (Czislowski et al., 2017). Two fragments of this PCR product were predicted to be generated after the restriction digestion, one with 102 bp and the other with 241.

To identify STR4 isolates, primers were designed to anneal to SNP-rich regions of the *SIX8* gene which are unique to the homolog “b,” only present in STR4 (Czislowski et al., 2017). Likewise, to detect R1 isolates, primers were designed to anneal to the polymorphic regions of the homolog “b” of the *SIX6* gene, found to be present only in R1 by Czislowski et al. (2017).

### PCR and Restriction Digestion Conditions

DNA material extracted from isolates of *Fusarium* was used as templates for the amplification tests in this study. DNA was extracted from 5 to 7-day-old monoconidial cultures, grown on half-strength potato dextrose agar (PDA, Difco Laboratories) at 25°C, using the DNeasy Plant Mini Kt (Qiagen) on the QIACube (Qiagen) following the manufacturer’s recommendations. Approximately 50–100 mg of mycelium were used as initial material in the extractions (amount recovered by scraping the medium surface of a 100-mm diameter standard Petri dish fully covered with mycelium).

For all amplifications, MyTaq^TM^ Red DNA Polymerase (Bioline) was used in 20 uL reactions according to the manufacturer’s recommendations. The primer and DNA template concentration in the final PCR reaction were 0.4 μM and 5 pg. uL^-1^, respectively. The following cycling conditions were used for all PCR: 95°C for 3 min, 30 cycles of 95°C for 15 s, and annealing temperature were primer-set dependant (see Table 1) for 15 s and 72°C for 10 s. In the cases of follow-up restriction digestions (for *SIX1* and *SIX13* products), no purification step of the PCR products is needed before enzymatic digestions. Restriction digestions for the enzymes HpyAV and EagI (New England Biolabs) were performed using 4 μL of the amplification product and 0.4 units (U) of enzyme in a total volume of 10 μL. The enzyme EagI is also known as Eco521. Star activity is a property exhibited by some restriction enzymes in which they show relaxed or inaccurate sequence recognition (Wei et al., 2008). HpyAV and EagI-HF have not been predicted to have star activity in the buffers recommended by the manufacturer^4^. If the recommended conditions are used as reported here, no restriction enzyme star activity is expected to occur. The restriction enzymes digestions were conducted at 37°C for 1 h and subsequently inactivated for 20 min at 65°C. A volume of 5 μL of amplification products and 10 μL restriction digestions were run on a 1.5% agarose gel and post-stained with ethidium bromide (1 μg mL^-1^).

The primer set that partially amplifies the translation elongation factor 1α gene (*TEF-1*α) was used in an additional PCR for all samples to confirm that each fungal DNA sample was amenable to amplification and control for false negatives (O’Donnell et al., 1998).

### Method Validation

To validate the diagnostic assays, the international standards as proposed in the “guidelines for the validation and verification of quantitative and qualitative test methods” were followed (National Association of Testing Authorities, 2018). These guidelines delineate particular requirements that a proposed method should meet to fulfill the purpose for its intended use (i.e., “fit-for-purpose”). As validation parameters, we included inter and intraspecific analytical specificity, sensitivity, robustness, repeatability, and reproducibility.

A total of 250 isolates of *Fusarium* spp. were screened in the validation tests. These included 16 different plant-associated *Fusarium* species in addition to 21 VCGs commonly associated with *Foc* (VCGs 120 to 01223).

For validation of assay specificity, we tested each primer set with 16 *Fusarium* species (Table 2). These *Fusarium* isolates were obtained from infected plant material in Australia. We also included in the validations nine different *formae speciales* of *F. oxysporum* and 21 different VCGs of *Foc*. Sensitivity was tested in a 10-fold serially diluted positive control within the range of 10 ng to 0.1 fg.μL^-1^ (Supplementary Table S1). Before the preparation of dilutions, DNA concentrations were measured in a Qubit fluorometer with the Quant-iT High-Sensitivity dsDNA Assay Kit (Thermo Fisher Scientific).

**Table 2:** Strains used for inter-specific validation of the diagnostic assays using the primer sets SIX1a_266 (TR4), SIX9_Foc/SIX10a_309 duplex (R4 VCG 0121), SIX13c_343 (R4 VCG 0121), SIX8b_206 (STR4) and SIX6b_210 (R1) and DNA extracted from *Fusarium* isolates obtained from infected plant material in Australia.

Code	Identification	Host
BRIP 61484a	*Fusarium acutatum*	*Solanum lycopersicum*
BRIP 53804a	*Fusarium ananatum*	*Ananas comosus*
BRIP 47260a	*Fusarium equiseti*	*Fragaria ananassa*
BRIP 26010	*Fusarium equiseti*	*Citrullus lanatus*
UQ6530	*Fusarium fujikuroi*	*Musa* sp. (Lady Finger)
UQ6540	*Fusarium fujikuroi*	*Musa* sp. (Lady Finger)
BRIP 61879a	*Fusarium meridionale*	Musa sp.
UQ6547	*Fusarium proliferatum*	*Musa* sp. (Lady Finger)
BRIP 63776	*Fusarium pseudocircinatum*	*Gossypium arboreum*
BRIP 61022a	*Fusarium pseudocircinatum*	*Mangifera indica*
BRIP 16558a	*Fusarium roseum* ‘Gibbosum’	*Passiflora edulis*
BRIP 53693a	*Fusarium roseum ‘*Gibbosum’	*Gossypium hirsutum*
BRIP 53695a	*Fusarium roseum* ‘Gibbosum’	*Rosa* sp.
UQ6659	*Fusarium sacchari*	*Musa* sp. (Lady Finger)
148	*Fusarium sacchari*	*Musa* sp. (Lady Finger)
212	*Fusarium sacchari*	*Musa* sp. (Lady Finger)
UQ6561	*Fusarium sacchari*	*Musa* sp. (Lady Finger)
UQ6562	*Fusarium sacchari*	*Musa* sp. (Lady Finger)
UQ6564	*Fusarium sacchari*	*Musa* sp. (Lady Finger)
UQ6567	*Fusarium sacchari*	*Musa* sp. (Lady Finger)
UQ6569	*Fusarium sacchari*	*Musa* sp. (Lady Finger)
UQ6576	*Fusarium sacchari*	*Musa* sp. (Lady Finger)
UQ6586	*Fusarium sacchari*	*Musa* sp. (Lady Finger)
UQ6588	*Fusarium sacchari*	*Musa* sp. (Lady Finger)
UQ6589	*Fusarium sacchari*	*Musa* sp. (Lady Finger)
UQ6590	*Fusarium sacchari*	*Musa* sp. (Lady Finger)
UQ6675	*Fusarium sacchari*	*Musa* sp. (Lady Finger)
BRIP 53261b	*Fusarium semitectum*	*Sorghum bicolor*
UQ6527	*Fusarium solani*	Musa sp. (Lady Finger)
UQ6609	*Fusarium solani*	*Musa* sp. (Lady Finger)
UQ6528	*Fusarium solani*	*Musa* sp. (Lady Finger)
UQ6610	*Fusarium solani*	*Musa* sp. (Lady Finger)
UQ6673	*Fusarium solani*	*Musa* sp. (Lady Finger)
UQ6548	*Fusarium solani/falciform*	*Musa* sp. (Lady Finger)
UQ6524	*Fusarium solani*/*falciform*	*Musa* sp. (Lady Finger)
BRIP 61517e	*Fusarium* sp.	*Persea americana*
BRIP 59719a	*Fusarium sterilihyphosum*	*Mangifera indica*
BRIP 52901a	*Fusarium tricinctum*	*Vitis vinifera*


*No amplification product was generated for any of the *Fusarium* isolates tested above.*

To test for assay robustness, two different *Taq* polymerases were used for the amplification of eight positive controls, in duplicate, in two different thermal cyclers. The DNA polymerases used were MyTaq^TM^ HS DNA polymerase (Bioline, MyTaq reaction buffer with 1 mM dNTPs, 3 mM MgCl_2_) and ThermoFisher Scientific *Taq* Polymerase [*Taq* Buffer with (NH_4_)_2_SO_4_], using reagent concentrations and cycling conditions as previously described. The robustness of the assays was tested by changing the manufacturer of the DNA polymerases. Different manufacturers of restriction enzymes were not used for the assays that included an additional restriction digestion step (*SIX1* and *SIX13*). The reason for that is based on reportedly different outcomes observed according to the type of polymerase (Schierwater and Ender, 1993), while the results obtained with restriction enzymes from different manufacturers are more consistent due to the intrinsic specificity of these enzymes.

For repeatability, six to eight positive controls from a variety of VCGs in six separate occasions were tested by the same operator for each primer set. For all assays, two negative controls were also included: one that contained a DNA sample that was not targeted by the primers and another that lacked DNA template. Reproducibility was tested by conducting separate assays with two different operators, on three different occasions, with the same set of samples. All tests included two technical replicates. Two previously reported conventional PCR methods were also compared with our assays: one utilized primers which amplify a region of the intergenic spacer (IGS) region of the nuclear ribosomal operon to detect TR4 (Dita et al., 2010); the other utilized primers developed from a Random Amplified Polymorphic DNA marker specific to *Foc* Race 4 (Lin et al., 2009).

## Results

Sequences from previously reported homologs of each *SIX* gene (Czislowski et al., 2017) were aligned and conserved regions within each race were identified as potential primer annealing sites. The alignment of different homologs of the genes *SIX1*, *SIX13*, *SIX8*, *SIX6* allowed the identification of exclusive sequences for TR4, R4 (VCG 0122), STR4 and R1, respectively. As *SIX10* was reported to be only present in R4 VCG 0121, and only one homolog has been reported (Czislowski et al., 2017), no alignment was needed prior to primer design targeting this gene.

Details on the presence of sufficient sequence polymorphisms that enabled race-specific primer design are described in the subsections dedicated to each *Foc* race below. All diagnostic assays were validated using DNA extracted from 38 isolates of *Fusarium* affiliated to 16 different species and 59 isolates of *F. oxysporum* obtained from healthy plant tissues of a range of plant species including, amongst others, *Asparagus officinalis*, *Citrullus lanatus*, *Cucumis melo*, *Euphorbia dallachyna*, *Musa* spp., *Phoenix* sp., *Solanum lycopersicum*, *Solanum tuberosum*, *Trianthema portulacastrum*, and *Zingiber officinale*. This included endophytes that were asymptomatic on their hosts and *F. oxysporum* classified into different *formae speciales*. A total of 150 *Foc* isolates affiliated to different races were used in our tests, including 32 TR4 isolates obtained in various countries (Tables 2–4).

**Table 3:** Intra-specific validation of the diagnostic assays using the primer sets SIX1a_266 (TR4), SIX9_Foc/SIX10a_309 duplex (R4 VCG 0121), SIX13c_343 (R4 VCG 0121), SIX8b_206 (STR4) and SIX6b_210 (R1) and DNA extracted from *Fusarium* isolates obtained from infected plant material in Australia.

Code	Identification	Host
1756	*Fusarium oxysporum*	*Phoenix* sp.
1755	*Fusarium oxysporum*	*Phoenix* sp.
WEED6	*Fusarium oxysporum*	*Trianthema portulacastrum*
WEED5	*Fusarium oxysporum*	*Euphorbia dallachyna*
WEED10	*Fusarium oxysporum*	*Rhynchosia minima*
BRIP 13106	*Fusarium oxysporum*	*Cucumis melo*
23732	*Fusarium oxysporum*	*Musa* sp.
23733-P	*Fusarium oxysporum*	*Musa* sp.
23733-W	*Fusarium oxysporum*	*Musa* sp.
BRIP 14928	*Fusarium oxysporum*	*Pisum sativum*
BRIP 16617	*Fusarium oxysporum*	*Trifolium repens*
BRIP 62618	*Fusarium oxysporum*	*Musa* sp. (DPM25)
BRIP 62577a	*Fusarium oxysporum*	*Musa* spp. (Cavendish)
GRS1058	*Fusarium oxysporum*	*Asparagus officinalis*
GRS585	*Fusarium oxysporum*	*Solanum lycopersicum*
GRS1054	*Fusarium oxysporum*	*Citrullus lanatus*
GRS1049	*Fusarium oxysporum*	*Solanum tuberosum*
GRS1034	*Fusarium oxysporum*	*Solanum tuberosum*
GRS1021	*Fusarium oxysporum*	*Solanum lycopersicum*
GRS1017	*Fusarium oxysporum*	*Asparagus officinalis*
GRS1008	*Fusarium oxysporum*	*Petroselinum crispum*
GRS1007	*Fusarium oxysporum*	*Citrullus lanatus*
GRS1002	*Fusarium oxysporum*	*Solanum lycopersicum*
FCC0778	*Fusarium oxysporum*	*Pinus patula*
FCC0776	*Fusarium oxysporum*	*Pinus patula*
CMH6015B	*Fusarium oxysporum*	*Citrullus lanatus*
CMH6008	*Fusarium oxysporum*	*Citrullus lanatus*
CMH6007	*Fusarium oxysporum*	*Citrullus lanatus*
CMH6002	*Fusarium oxysporum*	*Citrullus lanatus*
CMH6001	*Fusarium oxysporum*	*Citrullus lanatus*
CMH5727A	*Fusarium oxysporum*	*Petroselinum crispum*
GRS942(3)	*Fusarium oxysporum*	*Citrullus lanatus*
GRS920	*Fusarium oxysporum*	*Ocimum basilicum*
GRS932	*Fusarium oxysporum*	*Solanum lycopersicum*
GRS897	*Fusarium oxysporum*	*Solanum lycopersicum*
GRS895	*Fusarium oxysporum*	*Solanum lycopersicum*
GRS894	*Fusarium oxysporum*	*Solanum lycopersicum*
GRS652	*Fusarium oxysporum*	*Solanum lycopersicum*
BRIP 63620	*Fusarium oxysporum* f. sp. *basilici*	*Ocimum basilicum*
BRIP 63545	*Fusarium oxysporum* f. sp. *basilici*	*Ocimum basilicum*
BRIP 63616	*Fusarium oxysporum* f. sp. *basilici*	*Ocimum basilicum*
BRIP 63616	*Fusarium oxysporum* f. sp. *basilici*	*Ocimum basilicum*
BRIP 62106	*Fusarium oxysporum* f. sp. *fragariae*	*Fragaria ananassa*
BRIP 13039	*Fusarium oxysporum* f. sp. *lycopersici*	*Solanum lycopersicum*
BRIP 53843	*Fusarium oxysporum* f. sp. *lycopersici*	*Solanum lycopersicum*
BRIP 5181	*Fusarium oxysporum* f. sp. *niveum*	*Citrullus lanatus*
BRIP 5177	*Fusarium oxysporum* f. sp. *niveum*	*Citrullus lanatus*
BRIP 5178	*Fusarium oxysporum* f. sp. *niveum*	*Citrullus lanatus*
BRIP 28044	*Fusarium oxysporum* f. sp. *passiflorae*	*Passiflora edulis*
BRIP 57641	*Fusarium oxysporum* f. sp. *tracheiphilum*	*Vigna unguiculata* subsp. *sesquipedalis*
BRIP 43336	*Fusarium oxysporum* f. sp. *vasinfectum*	*Gossypium hirsutum*
BRIP 43344	*Fusarium oxysporum* f. sp. *vasinfectum*	*Gossypium hirsutum*
BRIP 43351	*Fusarium oxysporum* f. sp. *vasinfectum*	*Gossypium hirsutum*
BRIP 43356	*Fusarium oxysporum* f. sp. *vasinfectum*	*Gossypium hirsutum*
BRIP 43365	*Fusarium oxysporum* f. sp. *vasinfectum*	*Gossypium hirsutum*
BRIP 63607	*Fusarium oxysporum* f. sp. *vasinfectum*	*Gossypium hirsutum*
BRIP 25374	*Fusarium oxysporum* f. sp. *vasinfectum*	*Gossypium hirsutum*
BRIP 43339	*Fusarium oxysporum* f. sp. *vasinfectum*	*Gossypium hirsutum*
BRIP 44986	*Fusarium oxysporum* f. sp. *zingiberi*	*Zingiber officinale*


*No amplification product was generated for any of the *F. oxysporum* isolates tested above.*

**Table 4:** Validation of the diagnostic assays using the primer sets SIX1a_266 (TR4), SIX9_Foc/SIX10a_309 duplex (R4 VCG 0121), SIX13c_343 (R4 VCG 0121), SIX8b_206 (STR4) and SIX6b_210 (R1) and DNA extracted from isolates from the *Fusarium oxysporum* f. sp. *cubense* (*Foc*) species complex infecting *Musa* spp.

Code	Race^∗^	VCG	Country	TR4-specific	R4 VCG 0121-	R4 VCG0122-	STR4-	R1-specific
			of origin	*SIX1*a_266	specific duplex	specific	specific	*SIX*6b_210
					SIX9_Foc and	SIX13c_343	*SIX8*b_206	
					*SIX10a*_309			
58625	TR4	01213/16	Indonesia	+	-	-	-	-
58671	TR4	01213/16	Indonesia	+	-	-	-	-
58686	TR4	01213/16	Malaysia	+	-	-	-	-
58688	TR4	01213/16	Malaysia	+	-	-	-	-
58712	TR4	01213/16	Malaysia	+	-	-	-	-
58715	TR4	01213/16	Malaysia	+	-	-	-	-
58732	TR4	01213/16	Malaysia	+	-	-	-	-
58734	TR4	01213/16	Malaysia	+	-	-	-	-
58750	TR4	01213/16	Malaysia	+	-	-	-	-
58754	TR4	01213/16	Malaysia	+	-	-	-	-
58760	TR4	01213/16	Malaysia	+	-	-	-	-
59047	TR4	01213/16	Indonesia	+	-	-	-	-
59049	TR4	01213/16	Indonesia	+	-	-	-	-
59072	TR4	01213/16	Indonesia	+	-	-	-	-
59094	TR4	01213/16	Indonesia	+	-	-	-	-
59127	TR4	01213/16	Indonesia	+	-	-	-	-
59132	TR4	01213/16	Indonesia	+	-	-	-	-
59136	TR4	01213/16	Indonesia	+	-	-	-	-
59150	TR4	01213/16	Malaysia	+	-	-	-	-
62765	TR4	01213/16	Indonesia	+	-	-	-	-
62922	TR4	01213/16	India	+	-	-	-	-
62963	TR4	01213/16	Taiwan	+	-	-	-	-
63144	TR4	01213/16	Indonesia	+	-	-	-	-
63160	TR4	01213/16	Indonesia	+	-	-	-	-
63181	TR4	01213/16	Indonesia	+	+	-	-	-
63184	TR4	01213/16	Indonesia	+	-	-	-	-
63188	TR4	01213/16	Indonesia	+	+	-	-	-
63199	TR4	01213/16	Indonesia	+	-	-	-	-
63203	TR4	01213/16	Indonesia	+	-	-	-	-
63211	TR4	01213/16	Indonesia	+	-	-	-	-
63213	TR4	01213/16	Indonesia	+	-	-	-	-
63246	TR4	01213/16	Indonesia	+	+	-	-	-
58666	R4	0121	Indonesia	-	+	-	-	-
58738	R4	0121	Malaysia	-	+	-	-	-
58741	R4	0121	Malaysia	-	+	-	-	-
59084	R4	0121	Indonesia	-	+	-	-	-
59104	R4	0121	Indonesia	-	+	-	-	-
59106	R4	0121	Indonesia	-	+	-	-	-
59165	R4	0121	Taiwan	-	+	-	-	-
62962	R4	0121	Taiwan	-	+	-	-	-
63220	R4	0121	Indonesia	-	+	-	-	-
59154	R4	0122	Philippines	-	-	+	-	-
62808	R4	0122	Philippines	-	-	+	-	-
62892	R4	0122	Philippines	-	-	+	-	-
62894	R4	0122	Philippines	-	-	+	-	-
62901	R4	0122	Philippines	-	-	+	-	-
39259	STR4	0129/11	Australia	-	-	-	+	-
40309	STR4	0129	Australia	-	-	-	+	-
40334	STR4	0129	Australia	-	-	-	+	-
42113	STR4	0129	Australia	-	-	-	+	-
42130	STR4	0120	Australia	-	-	-	+	-
42131	STR4	0129	Australia	-	-	-	+	-
42134	STR4	0129	Australia	-	-	-	+	-
42135	STR4	0129	Australia	-	-	-	+	-
42186	STR4	0129	Australia	-	-	-	+	-
44012	STR4	0120	Australia	-	-	-	+	-
44027	STR4	0120	Australia	-	-	-	+	-
44073	STR4	01211	Australia	-	-	-	+	-
58610	STR4	0120/15	Canary Islands	-	-	-	+	-
58614	STR4	0120	Canary Islands	-	-	-	+	-
58620	STR4	0120	Indonesia	-	-	-	+	-
59052	STR4	0120/15	Indonesia	-	-	-	+	-
59093	STR4	0120/15	Indonesia	-	-	-	+	-
59162	STR4	0120	South Africa	-	-	-	+	-
59163	STR4	0120	South Africa	-	-	-	+	-
59787	STR4	0120/15	South Africa	-	-	-	+	-
59791	STR4	0120	South Africa	-	-	-	+	-
62581	STR4	0129	Australia	-	-	-	+	-
63532	STR4	0120	Australia	-	-	-	+	-
63615	STR4	0129	Australia	-	-	-	+	-
58637	R1^∗∗^	0126	Indonesia	-	-	-	+	-
58657	R1^∗∗^	0126	Indonesia	-	-	-	+	-
59044	R1^∗∗^	0126	Indonesia	-	-	-	+	-
59062	R1^∗∗^	0126	Indonesia	-	-	-	+	-
59152	R1^∗∗^	0126	Philippines	-	-	-	+	-
59153	R1^∗∗^	0126	Philippines	-	-	-	+	-
59161	R1^∗∗^	0126	Papua New Guinea	-	-	-	+	-
63200	R1^∗∗^	0126	Indonesia	-	-	-	+	-
42102	R1	01220	Australia	-	-	-	-	+
42174	R1	01220	Australia	-	-	-	-	+
42177	R1	01220	Australia	-	-	-	-	+
58617	R1	01218	Indonesia	-	-	-	-	+
58627	R1	01218	Indonesia	-	-	-	-	+
58680	R1	01217	Malaysia	-	-	-	-	+
58681	R1	01217	Malaysia	-	-	-	-	+
58683	R1	01217	Malaysia	-	-	-	-	+
58691	R1	01217	Malaysia	-	-	-	-	+
58698	R1	01217	Malaysia	-	-	-	-	+
58700	R1	01218	Malaysia	-	-	-	-	+
58710	R1	01217	Malaysia	-	-	-	-	+
58722	R1	0123	Malaysia	-	-	-	-	+
58723	R1	01217	Malaysia	-	-	-	-	+
58737	R1	0123	Malaysia	-	-	-	-	+
58742	R1	01217	Malaysia	-	-	-	-	+
58778	R1	0123	Philippines	-	-	-	-	+
58811	R1	0123	Thailand	-	-	-	-	+
59051	R1	0123	Indonesia	-	-	-	-	+
59071	R1	01218	Indonesia	-	-	-	-	+
59109	R1	01218	Indonesia	-	-	-	-	+
59145	R1	01217	Malaysia	-	-	-	-	+
59147	R1	01218	Malaysia	-	-	-	-	+
62542	R1	0123	Indonesia	-	-	-	-	+
62890	R1	0123	Philippines	-	-	-	-	-
63162	R1	0123	Indonesia	-	-	-	-	+
63175	R1	01218	Indonesia	-	-	-	-	+
63236	R1	01218	Indonesia	-	-	-	-	+
63581	R1	01220	Australia	-	-	-	-	+
63582	R1	01220	Australia	-	-	-	-	+
63583	R1	01220	Australia	-	-	-	-	+
63584	R1	01220	Australia	-	-	-	-	+
63585	R1	01220	Australia	-	-	-	-	+
63586	R1	01220	Australia	-	-	-	-	+
40176	R1	0125	Australia	-	-	-	-	+
40188	R1^#^	0125	Australia	-	-	-	-	+
42125	R1^#^	0124	Australia	-	-	-	-	-
42190	R1^#^	0124	Australia	-	-	-	-	-
44010	R1^#^	0125	Australia	-	-	-	-	+
44013	R1^#^	0128	Australia	-	-	-	-	+
44014	R1^#^	0125	Australia	-	-	-	-	+
44015	R1^#^	0128	Australia	-	-	-	-	+
44479	R1^#^	0128	Australia	-	-	-	-	+
44480	R1^#^	0128	Australia	-	-	-	-	+
44614	R1^#^	0128	Australia	-	-	-	-	+
58692	R1^#^	0125	Malaysia	-	-	-	-	+
58693	R1^#^	0124/5	Malaysia	-	-	-	-	+
58774	R1^#^	0124/5	Mexico	-	-	-	-	-
58788	R1^#^	0125	Thailand	-	-	-	-	+
58790	R1^#^	0124	Thailand	-	-	-	-	+
58813	R1^#^	0124/22	Uganda	-	-	-	-	+
59023	R1^#^	0124	Brazil	-	-	-	-	+
59033	R1^#^	0124	India	-	-	-	-	+
59036	R1^#^	0125	India	-	-	-	-	+
59087	R1^#^	0124	Indonesia	-	-	-	-	-
59788	R1^#^	0125	India	-	-	-	-	+
62794	R1^#^	0124/5	Philippines	-	-	-	-	+
62924	R1^#^	0125	India	-	-	-	-	+
62947	R1^#^	0124	Uganda	-	-	-	-	+
62950	R1^#^	0124/5	Brazil	-	-	-	-	+
62952	R1^#^	0125	Brazil	-	-	-	-	+
62961	R1^#^	0124	India	-	-	-	-	+
63531	R1^#^	0124	Australia	-	-	-	-	+
63537	R1^#^	0124	Australia	-	-	-	-	+
63600	R1^#^	0124/5	Australia	-	-	-	-	+
58624	Unknown	01219	Indonesia	-	-	-	+	-
58634	Unknown	01219	Indonesia	-	-	-	+	-
58635	Unknown	01219	Indonesia	-	-	-	+	-
58636	Unknown	01219	Indonesia	-	-	-	+	-
59037	Unknown	01212	India	-	-	-	-	+
59115	Unknown	01219	Indonesia	-	-	-	+	-
59170	Unknown	01222	Uganda	-	-	-	-	-
62955	Unknown	01212	India	-	-	-	-	+
63186	Unknown	01219	Indonesia	-	-	-	+	-
63187	Unknown	01219	Indonesia	-	-	-	+	-


*The symbols ‘+’ and ‘-’ represent positive and negative results, respectively.*

*^∗^The race structure used here was based in the literature (Rep, 2005; Moore et al., 1993; Ploetz, 2004; Bentley et al., 1995, 1998; Katan and Di Primo, 1999; Gerlach et al., 2000; Jones, 2000; Groenewald et al., 2006).*

*^∗∗^The race of VCG 0126 is considered equivocal as isolates are phenotypically and genetically similar to race 4 (e.g., ability to synthesize odorous aldehydes on media) (Moore et al., 1991; Dale et al., 2017). Since the ability of VCG 0126 to infect Cavendish has not been supported by solid evidence, we considered here as race 1 based its currently known host range (Pérez Vicente et al., 2014).*

*^#^These VCGs have been sometimes classified as R1/R2. However, limited evidence suggests its ability to cause disease in the Bluggoe subgroup.*

### Diagnostic Assay to Detect TR4 Using PCR and Restriction Digestion

**FIGURE 1:**
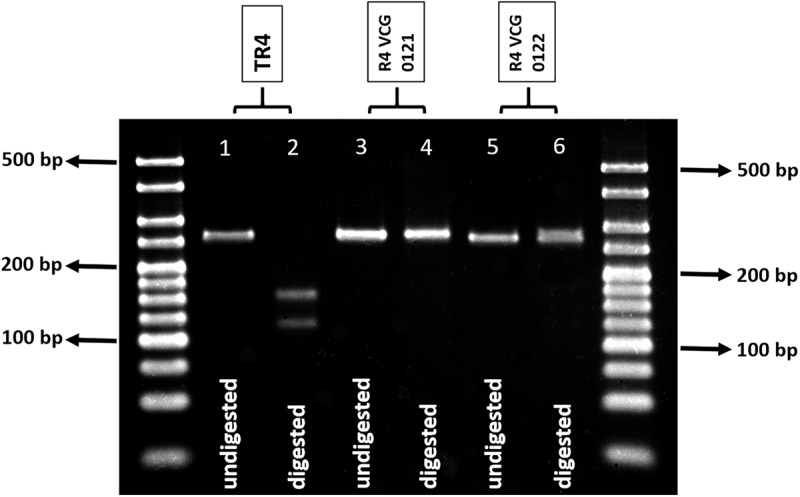
Agarose gel (3%) showing undigested and digested 266-bp amplification products obtained with the primer set (SIX1a_266) designed to target the *SIX* gene homolog present exclusively in TR4 (*SIX1*a). Ladder used: 25 bp Bioline Hyperladder. Lane 1, undigested amplicon from TR4 isolate (5 μL); lane 2, HpyAV-treated amplicon from TR4 isolate (10 μL); lane 3, undigested amplicon from R4 VCG 0121 isolate (5 μL); lane 4, HpyAV-treated amplicon from R4 VCG 0121 isolate (10 μL); lane 5, undigested amplicon from R4 VCG 0122 isolate (5 μL); and lane 6, HpyAV-treated amplicon from R4 VCG 0122 isolate (10 μL).

Sequences of the *SIX1* homologs “a” to “i” were aligned and the primer set SIX1a_266 was designed to anneal to regions that were conserved within homologs “a,” “b,” and “c,” and were exclusive to TR4, R4-VCG 0121, and R4-VCG 0122, respectively (Table 1). These primers flanked a recognition site of the enzyme HpyAV, which is only present in the *SIX1* gene homolog “a,” which is unique to TR4 (Czislowski et al., 2017). Figure 1 shows amplification products for TR4 isolates and HpyAV-digested fragments. The amplified product of 266 bp was digested into two fragments with the enzyme HpyAV, one with 124 bp and the other with 142 bp (Figure 1). The 266-bp amplification product is also obtained for R4-VCG 0121 and R4-VCG 0122; however, this product is not digested by HpyAV in strains associated with these VCGs (Figure 1). No false positives or false negatives were obtained for other *Fusarium* species, *F. oxysporum* strains, *formae speciales* of other *F. oxysporum*, or races tested (Tables 2–4). This assay was validated and proven to be repeatable, robust, and specific. The limit of detection is 1 pg.μL^-1^ (Supplementary Table S1).

### Diagnostic Assay to Detect R4-VCG 0121 Using a Duplex PCR

**FIGURE 2:**
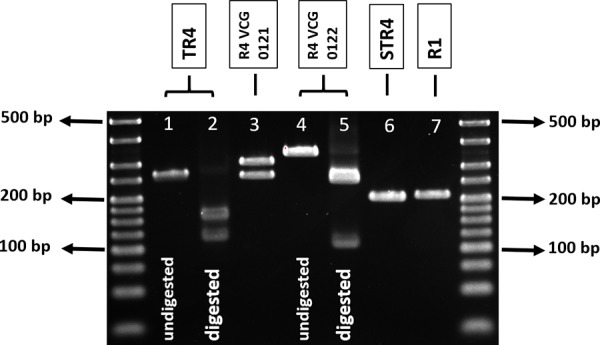
Agarose gel (3%) showing amplification products obtained with primers designed to target different races and VCGs of *Fusarium oxysporum* f. sp. *cubense*. Ladder used: 25 bp Bioline Hyperladder. Lane 1, undigested 266 bp *SIX1* gene amplicon (5 μL); lane 2, TR4-specific HpyAV-treated 266 bp *SIX1* gene amplicon (10 μL), showing 124 and 142 bp digests; lane 3, R4 VCG 0121-specific 309 and 260 bp amplicons from duplex PCR of the genes *SIX10* and *SIX9* (5 μL), respectively; lane 4, undigested 343 bp *SIX13* gene amplicon (5 μL); lane 5, R4 VCG 0122-specific EagI-treated 343 bp *SIX13* gene amplicon, showing 102 and 241 digests (10 μL); lane 6, STR4-specific 206 bp *SIX8* gene amplicon (5 μL); and lane 7, R1-specific *SIX6* 210 bp gene amplicon (5 μL).

The primer set which targets the gene *SIX10* in VCG 0121 (SIX10a_309) generated the expected amplification product in nine isolates of *F. oxysporum* that were asymptomatic endophytes in plant genera distinct to *Musa* (data not shown). For this reason, we developed a duplex PCR as the diagnostic assay for the detection of VCG 0121, which then included primers that target the homolog “a” of the gene *SIX9*. According to currently available data, this gene homolog seems to be present in *Foc* but absent in other *formae speciales* of *F. oxysporum* (Czislowski et al., 2017; Tables 3, 4). Two amplicons with the expected lengths were obtained using the primer sets SIX9_Foc and SIX10a_309, with 260 and 309 bp, respectively (Figure 2). The resultant duplex PCR has a limit of detection of 0.1 ng.μL^-1^ and met most validation criteria (Tables 2–4 and Supplementary Table S1). Four of 32 TR4 isolates from Indonesia tested positive for this duplex-PCR assay (Table 4), inferring the presence of *SIX10* in these isolates. The limit of detection was 0.01 ng.μL^-1^ (Supplementary Table S1).

### Diagnostic Assay to Detect R4-VCG 0122 Using PCR and Restriction Digestion

This assay was designed to target the gene *SIX13* and flanked a R4-VCG 0122-specific recognition site for the restriction enzyme Eagl (Table 1). The expected 343 bp product was amplified and, after digestion with the enzyme EagI, expected fragments of 102 and 241 bp were obtained (Figure 2). The assay using the primer set SIX13c_343 followed by an EagI-restriction digestion was specific, with no false negatives or false positives detected (Tables 3–5). The limit of detection of this assay was 0.01 g.μL^-1^ (Supplementary Table S1). An example of a gel showing amplification products of *SIX13* obtained with a range of DNA template concentrations is shown is Supplementary Figure S1. All validation criteria of repeatability and robustness were met.

**Table 5:** List of strains tested with primer sets reported in previous studies, which target TR4 and/or R4 strains and produced false positive results.

Code	Identification	Host	Country	TR4 primer	R4 primer
				set FocTR4^a^	set Foc1/2^b^
BRIP 62577a	*Fusarium oxysporum*	*Musa* sp.	Australia	+	-
GRS1054	*Fusarium oxysporum*	*Citrullus lanatus*	Australia	-	+
CMH6002	*Fusarium oxysporum*	*Citrullus lanatus*	Australia	-	+
BRIP 59052	*Foc* STR4 (VCG 0120/15)	*Musa* sp.	Indonesia	+	+
BRIP 43336	*Fusarium oxysporum* f. sp. *vasinfectum*	*Gossypium hirsutum*	Australia	+	+
BRIP 43344	*Fusarium oxysporum* f. sp. *vasinfectum*	*Gossypium hirsutum*	Australia	+	+
BRIP 43365	*Fusarium oxysporum* f. sp. *vasinfectum*	*Gossypium hirsutum*	Australia	+	+


*The symbols ‘+’ and ‘-’ represent positive and negative results, respectively.*

*^*a*^FAOSTAT (2018) and ^*b*^Lin et al. (2009).*

### Diagnostic Assay to Detect STR4 Using PCR

The primer set SIX8b_206 was designed to amplify exclusively STR4 isolates (Table 1). A 206 bp product was amplified for all tested STR4 isolates (Figure 2) and no false positives were obtained (Tables 2–4). Amplification products with the expected sizes were also obtained for isolates belonging to the VCGs 01219 (whose race is still undetermined) and VCG 0126 (classified as R1) (Table 4). This assay was proven to be repeatable, reproducible, robust, sensitive and specific. The limit of detection was 0.01 ng.μL^-1^ (Supplementary Table S1).

### Diagnostic Assay to Detect R1 Using PCR

The primer set SIX6b_210 was designed to detect exclusively R1 isolates (Table 1). The primers generated the expected 210 bp amplicon for most R1-associated VCGs (60 isolates out of 65, Figure 1 and Table 4). Three strains classified as R1 did not generate the expected PCR product, with one of them belonging to the VCG 0123 (BRIP 62890), three to the VCG 0124 (BRIPs 42125, 42190, and 59087), and the other to the VCG 0124/5 (BRIP 58774) (Table 4). The test was shown to be repeatable, robust, reproducible, and the limit of detection was 0.1 ng. uL^-1^ (Supplementary Table S1).

Although all primer sets generated amplicons using DNA templates at a concentration of 0.5 ng. μL^-1^ in the PCR reaction for most isolates, some false negatives were observed at this particular concentration. Thus, we recommend using DNA concentrations between 50 pg. μL^-1^ and 5 pg. μL^-1^ in the PCR reaction as these concentrations worked consistently well in the assays conducted to determine the limit of detection (Supplementary Table S1).

### Comparison With Previously Reported Molecular Diagnostics Methods to Detect R4

We confirmed unpublished results obtained by the Department of Agriculture and Fisheries (Henderson J., unpublished), which revealed that the primers FocTR4, previously reported to detect TR4 (Dita et al., 2010), tested positive for one endophytic strain isolated from an asymptomatic banana plant grown in Northern Queensland, Australia (BRIP62577, Table 5). Furthermore, the same primer set and the Foc1/2 primer set reported by Lin et al., 2009 tested positive for one STR4 and three *F. oxysporum* f. sp. *vasinfectum* isolates (BRIP 59052, 43336, 43344, and 43365, Table 5). The Foc1/2 primer set also produced amplification products for two endophytic strains of *F. oxysporum* colonizing watermelon plants (*Citrullus lanatus*) (GRS1054 and CMH6002, Table 5). As the Foc1/2 primer set cannot distinguish between TR4 and STR4, it is expected that amplicons for STR4 strains are produced, such as for BRIP 59052 (Table 5).

## Discussion

This study supports the utilization of *SIX* genes as targets for molecular diagnostics for races of *Foc*. The results demonstrate that for each race, specific conserved *SIX* gene sequences can be identified especially for TR4, R4-VCG 0122, and STR4. Sufficient SNPs across *SIX* gene homologs enabled the differentiation of TR4, R4-VCG 0121, R4-VCG 0122, STR4, and R1 strains through conventional PCR using primers targeting polymorphic regions of the genes *SIX1*, *SIX9*/*SIX10*, *SIX13*, *SIX8*, and *SIX6*, respectively. However, only a small number of SNPs was present to distinguish TR4 and R4-VCG 0122 from other races and VCGs. Therefore, following the PCRs targeting *SIX1* and *SIX13*, a restriction digestion step with the enzymes HpyAV and EagI was required to detect the specific homologs present in TR4 and R4 VCG 0122, respectively. Overall, our validation experiments revealed that our assays are specific, sensitive, robust and repeatable.

**FIGURE 3:**
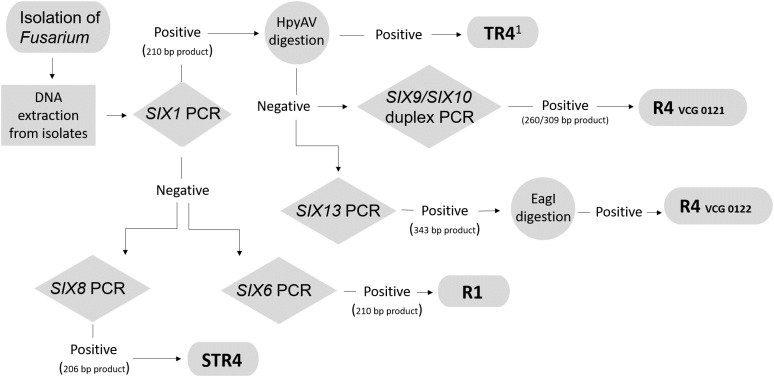
Proposed molecular diagnostic workflow to distinguish races and vegetative compatibility groups (VCGs) of *F. oxysporum* f. sp. *cubense*. based on a conventional PCR targeting the *SIX* genes. TR4^1^ has been proposed to be reclassified as *Fusarium odoratissimum* (Maryani et al., 2019).

Strains with VCG 0122 have been recently reported to infect the Cavendish cultivar Grande Naine (AAA) in the Davao del Norte area in the Philippines between 2006 and 2007 (Mostert et al., 2017), but Maryani et al. (2019) reported that these strains were not able to infect Cavendish in glasshouse trials. These contradictory results may stem from the low aggressiveness of these strains, which may not infect plants under artificial conditions. Some factors influencing the success of the infections in the field may not have been known and taken into account. The primer set SIX1a_266 generates amplicons for not only TR4, but also the R4 VCGs 0121 and 0122. Therefore, the primer sets SIX10a_309 and SIX13c_343 were additionally designed to detect exclusively R4 VCGs 0121 and 0122, respectively. Other PCR primers that can detect VCG 0121 and TR4 have been reported by Lin et al. (2009), but they cannot discriminate between TR4 and STR4 isolates. In the study by Czislowski et al. (2017) within *Foc*, only one homolog of the gene *SIX10* was identified and it was present exclusively in VCG 0121; consequently *SIX10* appeared to be a suitable specific target for VCG 0121. The *SIX10* gene is however, present in other *F. oxysporum* strains, as we observed from amplification products using isolates other than *Foc*. This led to the use of the additional primer set SIX9_Foc, which targets a gene homolog of *SIX9* previously shown to be present in all *Foc* (Czislowski et al., 2017). Despite the reported monophyletic nature of TR4 isolates (Ordonez et al., 2015; Maryani et al., 2019), our results indicate that there is some level of variability associated with the *SIX* gene profiles of these isolates given that amplicons of *SIX10* in the duplex PCR were observed in 12.5% of the TR4 isolates tested (4 out of 32). However, this finding does not affect the use of our diagnostic toolkit. For example, if Fusarium wilt symptoms are reported in a Cavendish plantation in a region where there is no record of TR4, the first molecular diagnostic method that should be conducted is a PCR using the SIX1a_266 primer set, followed by restriction digestion with the HpyAV enzyme. This is important given the increased risks associated with a TR4 incursion compared to the other races. If the expected 124 and 142 bp digestion products are obtained after the treatment with HpyAV, there is strong evidence to suggest that the isolate is TR4. An amplification reaction with primers targeting the gene *SIX9* should be run simultaneously with the primer set SIX1a_266 to verify whether the isolate is *Foc*. If this is confirmed, the isolate may be considered TR4. DNA from isolates with VCGs 0121 and 0122 will also generate an amplification product with this primer set but, because they lack the HpyAV restriction site, the 266-bp product will not be digested into two fragments. If this is the case, then the next step would be either to use the duplex PCR to verify whether it detects R4 VCG 0121, or the SIX13c_343 primer set to identify R4 VCG 0122. We advise that a PCR targeting the *SIX9* gene should be conducted in parallel with the race-specific PCR assay to confirm that the tested isolate is a *Foc* and not an endophytic *Fusarium* strain that shares the same *SIX* gene homolog with a pathogenic strain. In Figure 3 we suggest a workflow for the use of the different primer sets designed in this study.

The primer set SIX8b_206 was designed to amplify exclusively STR4 isolates. Amongst a wide range of strains tested, 24 STR4 isolates from the eight different VCGs considered to be STR4 were included in the assays (Table 4). A 206 bp product was amplified for all tested STR4 isolates and no false positives were obtained (Table 2). Amplification of products with the expected size were also obtained for isolates belonging to the VCGs 01219 and 0126, whose races are either not determined or still controversial. The race associated with the VCG 01219 has not been determined yet. However, our findings corroborate those of other studies that documented strains which belong to the VCG 01219 have the same *SIX* gene profile as all the other VCGs commonly associated with STR4 (Czislowski et al., 2017). In addition, despite VCG 0126 having been associated with R1 given its host range, our results are in agreement with reports suggesting closer genetic relatedness of VGC 0126 to isolates belonging to VCGs associated with STR4 than those associated with R1 (Bentley et al., 1998; Groenewald et al., 2006; Czislowski et al., 2017).

Although the expected results were obtained for most strains tested with the *SIX6* primers designed to amplify R1, five R1 isolates did not show the expected amplification product. Three of them belong to VCG 0124 and one to VCG 0123. A possible reason is the polyphyletic nature of R1 strains (Ordonez et al., 2016), which complicates the design of specific primers for this race. A recent phylogenetic analysis of *Foc* using strains isolated in the Indonesian center of origin revealed that diversity among genetic lineages of *Foc* is higher than previously anticipated (Maryani et al., 2019). Identification and phylogeny of these isolates were based on the genes encoding the translation elongation factor-1alpha (*tef1*), the RNA polymerase II largest submunit (*rpb1*), and the RNA polymerase II second largest subunit (*rpb2*), leading to the formal description of nine independent lineages (Maryani et al., 2019). TR4 isolates belong to the same lineage and were proposed to be reclassified as *F. odoratissimum* (Maryani et al., 2019). A previous attempt to design a real-time PCR specific for *Foc* R1 isolates has been reported but only four isolates belonging to this race were included and no VCG information was given (Yang et al., 2015). The distribution of putative effectors in natural and agro-ecosystems suggests that *SIX6*, which was chosen in this study to detect R1, may have been horizontally transferred across *F. oxysporum* strains (Rocha et al., 2016).

Pathogenicity genes have been common targets for molecular diagnostic assays of plant diseases (Loreti and Gallelli, 2002; Zaccardelli et al., 2007; Serdani et al., 2013). In fungi, the most typical avirulence factors are secreted small proteins (less than 200 amino acids) (Rep, 2005). Disease resistance is often triggered when the host’s innate immune system identifies these proteins (Kim et al., 2016). However, it is not unusual that fungi adopting endophytic lifestyles also harbor genes that encode these proteins (Kim et al., 2016). The occurrence of putative effector genes has been investigated in strains of the *F. oxysporum* species complex isolated from asymptomatic plants occurring in natural environments (Rocha et al., 2016; Jelinski et al., 2017). The genes *PDA1*, *PELD*, *SGE1*, and *SIX* were evaluated because of their involvement in pathogenicity and functional diversity (Rocha et al., 2016). From these putative effector genes, *SIX* genes were reported to be generally less prevalent, which suggests that they may not have a crucial function in natural populations of *F. oxysporum* (Rocha et al., 2016). *SIX* genes have already been used as targets for molecular diagnostics, as in the case of a loop-mediated isothermal amplification assay targeting the *SIX3* gene of *F. oxysporum* f. sp. *lycopersici* (Ayukawa et al., 2017). The proposed method claims to detect point mutations and distinguish race 3 strains from other races of *F. oxysporum* f. sp. *lycopersici* (Ayukawa et al., 2017). Furthermore, a previous study detected enough sequence variation in the *SIX8* gene to design a primer set which distinguished R4 strains from races 1 and 2 and another set which differentiates STR4 from TR4 by the presence of an amplification product only in STR4 (Fraser-Smith et al., 2014). The main issue with this approach is its reliance on the absence of a band to test positive for TR4, which is not acceptable as a robust diagnostic test.

A range of molecular diagnostics methods has been previously proposed to distinguish R4 from other races of *Foc*. Lin et al. (2009) proposed primers to detect R4 isolates based on a random amplification of polymorphic DNA (RAPD) product that was unique to R4 strains. However, their validation tests included only seven reference *Foc* isolates from other races (two R1 isolates, two R2, and three STR4 isolates). This primer set was further tested in at least two other studies. Dita et al., 2010 found that these primers amplified nine different *Foc* VCGs, including several that included strains which are not classified as R4, such as the ones belonging to the VCG 01210 that typically includes R1 isolates (Dita et al., 2010). Another study reported that the primers developed by Lin et al. (2009) tested positive for endophytic strains isolated from healthy banana plants and R1 isolates (Magdama, 2017). This primer set is also unable to distinguish STR4 from TR4 isolates, which is a major drawback for regions where STR4 is endemic and that still have no record of TR4. Alternative primers targeting a ribosomal IGS have been developed and found to be quite reliable (Dita et al., 2010). This region was chosen as it was deemed more polymorphic than others and more suitable as a sensitive diagnostic test due to the multi-copy nature of this region (Fourie et al., 2009; Dita et al., 2010), which was confirmed by our tests (Supplementary Table S1). Nonetheless, the primers FocTR4 which were proposed by Dita et al., 2010 were tested in another study which included endophytic strains isolated from healthy banana plants (Magdama, 2017). An endophytic *F. oxysporum* strain which was obtained from Gros Michel roots tested positive although it was isolated from an asymptomatic plant grown in a region where TR4 is absent (Magdama, 2017). In our study, we also obtained false positives for one endophytic strain isolated from an asymptomatic banana plant, one STR4 and three *F. oxysporum* f. sp. *vasinfectum* isolates with primers FocTR4 (Dita et al., 2010; Table 5). It is possible that there is a higher risk of obtaining false positives when targeting core genomic regions, as genetically related strains may contain the same sequence and still differ in pathogenicity. Primers designed to amplify genes or sequences associated with pathogenicity may be more specific in a diagnostic assay; however, their sensitivity may be lower compared to other regions that are often present as multiple copies in the genome, such as ribosomal regions (Black et al., 2013).

It is important to point out that our molecular diagnostics toolkit was designed and validated to be used only with DNA extracted from pure cultures of *F. oxysporum* as templates. Thus, we strongly discourage the use of our assays beyond the parameters validated here. Further tests with the use of DNA extracted from infected plant material are needed and require extensive validation. The ubiquitous nature of *F. oxysporum* existing in plant tissue as saprophytic and/or endophytic strains with apparent diverse and fickle genotypes renders the development of molecular based diagnostic assays direct from plant tissue, challenging. This would be the main reason why we would be hesitant to convert our conventional PCR assays into loop-mediated isothermal amplification (LAMP) assays, which are mainly used either in crude samples extracts or DNA extracted directly from tissues rather than cultures (Zhang et al., 2014). LAMP is a technique that is used for amplifying a specific segment of DNA under isothermal conditions through the strand-displacing *Bst* DNA polymerase (Zhang et al., 2014). This tool is commonly advocated as being sensitive, low cost and mobile; however, the combined price of the needed equipment, kits and reagents is considerable. In addition, some expertise is needed for the interpretation of the results in the field, suitable controls are also necessary, and results need to be confirmed in a centralized laboratory through an alternative effective diagnostic assay. Our results also suggest that there is not enough variability in the *SIX* gene sequences across *Foc* races to allow the design of four primers that recognize six distinct regions. For example, our assay for TR4 relies on the presence of two SNPs in the *SIX1* homolog “a,” which is exclusive to TR4 strains and is part of the recognition site of the HpyAV enzyme.

The availability of specific, sensitive, and robust diagnostic assays to identify plant pathogens is vital for the early detection and further containment or eradication of plant diseases. Accurate identification of the race or VCG of *Foc* using a sensitive, robust, user-friendly, and accessible assay by laboratories in any part of the world can provide reliable diagnostics to growers. This would also assist local governments to take suitable control measures to prevent threats to food security or economic losses to the banana industry.

## Author Contributions

JH, EA, EC, and AD conceived the study. JH, AD, and LC designed the experiments. EA and EC provided the sequences for primer design. CO, VR-F, and LC carried out the experiments. LC and AD drafted the manuscript. All authors contributed to the final manuscript.

## Conflict of Interest Statement

The authors declare that the research was conducted in the absence of any commercial or financial relationships that could be construed as a potential conflict of interest.

## Footnotes

1 https://www.ncbi.nlm.nih.gov/nuccore/

2 https://www.ebi.ac.uk/Tools/msa/clustalo/

3 https://www.ncbi.nlm.nih.gov/tools/primer-blast

## Supplementary Material

Figure S1 Example of agarose gel showing the limit of detection of amplification products obtained with the primer set SIX13c_343 within a range of DNA template concentrations.

Table S1 Limit of detection of primer sets SIX1a_266, SIX6b_210, SIX8b_206, SIX9_Foc, SIX10a_309, SIX13c_343, and the previously published primers Foc-TR4 (Dita et al., 2010), and EF (O’Donnell et al., 1998) within the DNA template concentration range of 10 ng⋅μL^-1^ to 0.1 fg⋅μL^-1^.
