# Supplementary figures and images for: Molecular Diagnostics of Banana Fusarium Wilt Targeting Secreted-in-Xylem Genes
Source: Front Plant Sci. 2019 May 31;10:547. doi: 10.3389/fpls.2019.00547 (PMC6554419; doi:10.3389/fpls.2019.00547)

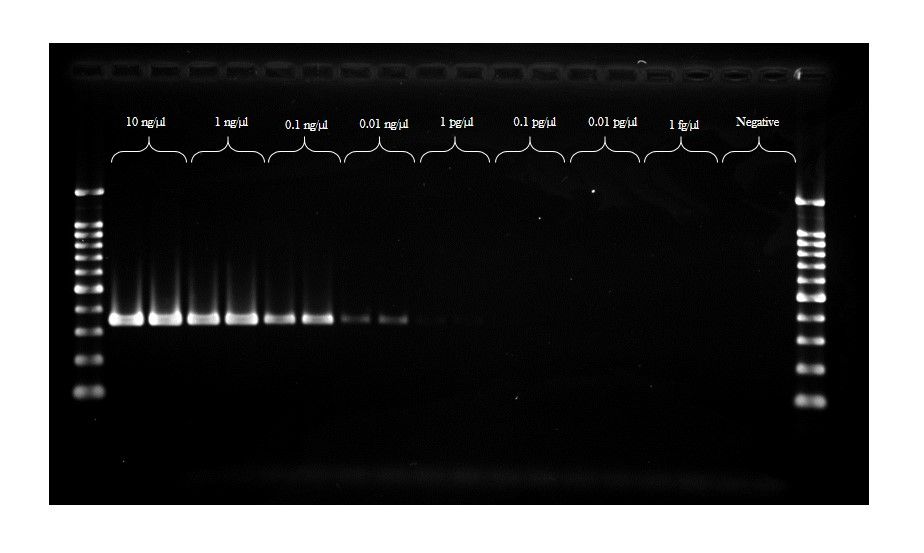

Supplement: Figure S1 — Example of agarose gel showing the limit of detection of amplification products obtained with the primer set SIX13c_343 within a range of DNA template concentrations. [file Image_1.JPEG]
